# The Role of Iron in Phytopathogenic Microbe–Plant Interactions: Insights into Virulence and Host Immune Response

**DOI:** 10.3390/plants12173173

**Published:** 2023-09-04

**Authors:** Sheo Shankar Pandey

**Affiliations:** 1 Life Sciences Division, Institute of Advanced Study in Science and Technology (IASST), Guwahati 781035, India; sshankar.pandey@iasst.gov.in; Tel.: +91-361-2270095 (ext. 216); 2 Citrus Research and Education Center (CREC), Department of Microbiology and Cell Science, Institute of Food and Agricultural Sciences, University of Florida, Lake Alfred, FL 33850, USA

**Keywords:** iron, iron homeostasis, iron uptake and metabolism, pathogens, phytopathogenic microbes, bacteria, iron in microbial virulence, plant–microbe interactions

## Abstract

Iron is an essential element required for the growth and survival of nearly all forms of life. It serves as a catalytic component in multiple enzymatic reactions, such as photosynthesis, respiration, and DNA replication. However, the excessive accumulation of iron can result in cellular toxicity due to the production of reactive oxygen species (ROS) through the Fenton reaction. Therefore, to maintain iron homeostasis, organisms have developed a complex regulatory network at the molecular level. Besides catalyzing cellular redox reactions, iron also regulates virulence-associated functions in several microbial pathogens. Hosts and pathogens have evolved sophisticated strategies to compete against each other over iron resources. Although the role of iron in microbial pathogenesis in animals has been extensively studied, mechanistic insights into phytopathogenic microbe–plant associations remain poorly understood. Recent intensive research has provided intriguing insights into the role of iron in several plant–pathogen interactions. This review aims to describe the recent advances in understanding the role of iron in the lifestyle and virulence of phytopathogenic microbes, focusing on bacteria and host immune responses.

## 1. Introduction

Iron is accredited as the most abundant element on earth and ranks as the fourth most abundant element in the earth’s crust. It can exist in various oxidation states, ranging from −2 to +6. The primordial ocean had an abundance of ferrous iron, but oxygenation of the earth’s environment led to its oxidation into the ferric form of iron. Iron commonly occurs in biological systems in either the +3 (ferric) or +2 (ferrous) oxidation states [1]. Ferric iron is abundant, relatively unreactive, and insoluble, while ferrous iron is scarce, reactive, and soluble. The stable nucleus, ligand binding property, and ability to catalyze redox reactions are some of the reasons why iron is an indispensable element in the genesis and evolution of life [1,2]. 

**Figure 1:**
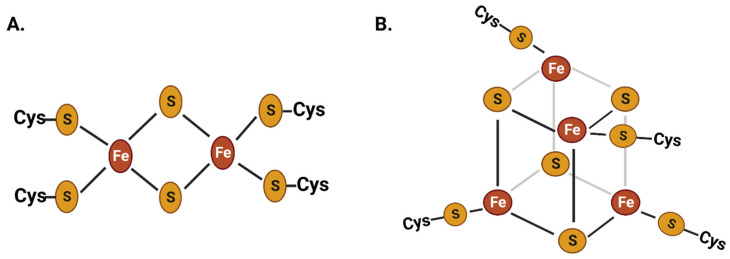
**Chemical structure of two of the most common iron–sulfur clusters:** the 2Fe-2S (**A**) and the 4Fe-4S (**B**) clusters. These iron–sulfur clusters have been present in life since the ancient stages of evolution. Iron is abundantly present in metalloproteins as part of these iron–sulfur clusters, which play a crucial role in cellular redox reactions.

Iron is an essential cofactor in cellular redox reactions due to its ability to transition between ferrous and ferric oxidation states with moderate oxidation potential and its broad range of ligand-binding capabilities [1,3]. The iron-bound proteins constitute around 50% of all metalloproteins in living beings. The abundance of iron sulfur proteins with [2Fe-2S] and [4Fe-4S] clusters in biological redox reactions is considered extremely ancient that incorporated in life at the early stage of evolution (Figure 1) [4,5,6,7]. Iron is abundantly distributed in various subcellular compartments, such as mitochondria, chloroplasts, lysosomes, Golgi complexes, nuclei, and nucleoli [8,9,10,11]. Iron metalloproteins play critical roles in a broad range of cellular and physiological reactions, including erythropoiesis, respiration, chloroplast/mitochondrial metabolism, host immunity, cell proliferation, and amino acid and nucleic acid metabolism. Additionally, iron plays a crucial role in regulating gene expression through various iron-associated transcription factors [12,13,14,15,16].

The acquisition of iron is imperative for essential subcellular redox reactions in nearly all forms of life, making the struggle for control over this resource a critical aspect of the evolutionary battle between microbial pathogens and their hosts. Most research investigating the role of iron in host–microbe interactions has focused on mammalian–pathogen associations. Hosts have evolved sophisticated strategies for withholding iron to restrict free-iron availability to colonizing pathogens. The ability to extract iron and adapt to low-iron environments inside hosts is crucial in determining the survival and virulence of several bacterial, protozoan, viral, and fungal pathogens on mammals [17,18,19,20,21,22,23,24]. Recent extensive studies have provided mechanistic insights into the significance of iron homeostasis in the virulence of phytopathogenic bacteria, fungi, and viruses on plants. Iron homeostasis in phytopathogenic microbes is regulated by complex regulatory networks that are fine-tuned by the involvement of multiple known and unknown factors. Research suggests a diverse and contrasting regulation of factors associated with iron homeostasis in closely related phytopathogenic bacteria [25,26,27]. This review aims to discuss a comprehensive overview of recent advances and understanding regarding the role of iron homeostasis in the lifestyle and virulence of phytopathogenic microbes. Furthermore, this review describes research progress on the regulation of iron-dependent plant host responses against microbial infections.

## 2. Mechanistic Insights into Phytopathogenic Bacterial Iron Homeostasis and Virulence

Host plants and phytopathogenic microbes have developed sophisticated strategies to regulate their own iron homeostasis, restricting the availability of this essential element to other organisms. In this section, we delineate the research advancements concerning various strategies employed by phytopathogenic microbes for iron acquisition, and elucidate their contribution to the expression of virulence.

### 2.1. Microbial Iron Acquisition and Virulence

**Figure 2:**
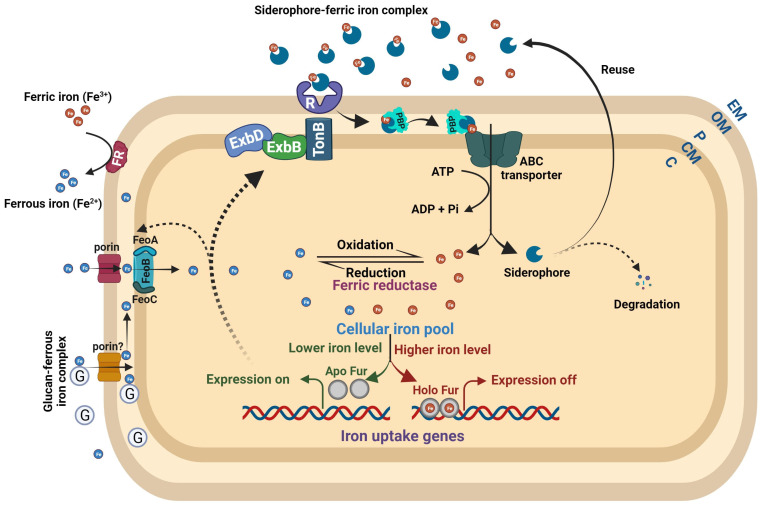
**Bacterial iron homeostasis.** The ferric and ferrous iron uptake pathways are independent in nature but markedly interdependent in regulation. Under high intracellular iron, the Holo Fur (Fur–Fe^2+^ complex) binds to the regulatory sites of iron uptake genes and turns off their expression. When intracellular iron levels are low, the Holo Fur releases the Fe^2+^ iron, and it turns into Apo Fur (Fur alone). The Apo Fur loses the ability to bind to regulatory sites, which makes the regulatory sites free from Fur and enables the expression of iron uptake genes. Bacteria synthesize ferric iron-chelating compounds, siderophores, and release them into the extracellular milieu to sequester ferric iron. The TonB-dependent outer membrane receptors recognize the Fe^3^+–siderophore complex, causing a conformational change in the plug domain of the receptor’s channel to internalize it. ExbB and ExbD energize TonB using an electrochemical charge gradient along the cytoplasmic membrane to release the Fe^3+^–siderophore complex into the periplasmic space. Further, periplasmic-binding proteins deliver the complex to the cognate ABC transporter to transport it into the cytoplasm. The ferrous iron is transported to the periplasm by Fe^2+^-specific porins. The glucan–Fe^2+^ complex can also bring ferrous iron to the periplasmic space. Further, the FeoB complex (FeoABC) transporter transports the ferrous iron to the cytoplasm. The outer membrane and cytoplasmic ferric reductases reduce ferric iron to ferrous iron at their respective places. Abbreviations: R = receptor; G = glucan; PBP = periplasmic-binding protein; EM = extracellular moiety; OM = outer membrane; P = periplasm; CM = cytoplasmic membrane; C = cytoplasm.

Bacteria encountering low iron levels either inside the host or under laboratory conditions typically produce and secrete siderophores, which scavenge ferric iron. Siderophore-mediated iron uptake is highly efficient, but requires the consumption of energy via ATP hydrolysis (Figure 2). Siderophores, which means “iron carriers” in Greek, are low-molecular-weight compounds secreted through exporters that usually belong to the major facilitator superfamily (MFS), ATP-binding cassette (ABC) superfamily, and resistance–nodulation–cell division (RND) superfamily transporters. In *Escherichia coli*, the enterobactin siderophore is secreted from the cytoplasm to the periplasm by the major facilitator EntS and then from the periplasm to the extracellular milieu via TolC, an outer membrane exporter of multidrug efflux pumps [28,29,30]. Siderophores exhibit high affinity to ferric iron and efficiently chelate iron in iron-depleted host environments or laboratory conditions. TonB-dependent outer membrane receptors, specific to ferric iron–siderophore complexes, recognize and internalize them into the periplasmic space with the involvement of the TonB-ExbBD complex. Further, periplasmic binding proteins (PBPs) deliver the ferric iron–siderophore complexes to the cognate ABC transporter, which transports them into the cytoplasm while ATP hydrolysis occurs (Figure 2) [31,32]. Specific porins transport ferrous iron from the extracellular milieu to the periplasm. The bacterial periplasmic and secreted β-cyclic glucan has the ability to sequester ferrous iron, which supports growth under low-iron conditions and protects cells against iron-induced toxicity under iron-replete conditions [33]. Several bacteria encode the FeoABC transporter for the import of ferrous iron from the periplasm to the cytoplasm (Figure 2) [34,35,36,37,38]. However, SitABCD in *Salmonella typhimurium,* YfeABCD in *Yersinia pestis*, SfuABC in *Serratia marcescens*, SitABCD in *Salmonella enterica*, and FbpABC in *Neisseria gonorrhoeae* are involved in ferrous iron import [39,40,41,42,43]. The FeoABC system is widely distributed among Gram-negative bacteria. Conversely, the SitABCD system is unique to certain pathogens such as *Salmonella typhimurium*, and it contributes to their virulence by effectively scavenging iron during infection. YfeABCD in *Yersinia pestis* acquires iron from host tissues, vital for pathogenicity. SfuABC, unique to *Serratia marcescens*, supports iron uptake from the environment, and the FbpABC transporter identified in *Neisseria gonorrhoeae* contributes to the bacterium’s growth and survival [39,40,41,42,43]. The outer membrane and cytoplasmic ferric reductases are responsible for reducing ferric iron to ferrous iron and maintaining the equilibrium between these two common oxidation states of iron (Figure 2) [44,45,46]. A more detailed understanding of bacterial iron uptake mechanisms can be found in reviews by Andrews et al. (2003), Krewulak and Vogel (2008), Chu et al. (2010), and Cornelis and Andrews (2010) [47,48,49,50]. In this section, we focus on describing the iron uptake systems and their roles in the virulence of phytopathogenic bacteria.

The enterobacterium *Dickeya dadantii* (formerly known as *Erwinia chrysanthemi*) causes soft rot disease on a broad host range of plants. *Dickeya dadantii* produces chrysobactin and achromobactin, two different types of siderophores that play an essential role in its full virulence on plants [51,52]. The enterobacterium *Dickeya dadantii* employs the type 2 secretion system (T2SS) to secrete the metal-binding protein IbpS, which exhibits high affinity for ferric and cupric ions. This substrate is essential for the virulence of *Dickeya dadantii* on plants and is conserved among numerous phytopathogenic microorganisms and fungi [53]. *Erwinia amylovora*, the pathogen responsible for the severe fire blight disease in apple and pear, produces the siderophore desferrioxamine (DFO) to sequester iron during the infection process. In addition to its role in supporting virulence by sequestering iron, DFO also plays a critical role in protecting the bacterium against the oxidative burst induced by the host plant’s defense response [54]. A recent study has reported that desferrioxamine (DFO) appears to be a significant virulence factor of *Erwinia amylovora* CFBP1430 in the low-iron state of apple flowers that is created after iron consumption by precolonized microorganisms [55]. *Erwinia carotovora* subsp. carotovora also produces the aerobactin and chrysobactin siderophores, but knocking out the genes encoding either of these siderophores does not affect the bacterium’s ability to macerate the potato tuber or develop aerial stem rot on potato [56,57]. *Pseudomonas syringae* pv. tabaci 6605, a foliar bacterial pathogen that causes wildfire disease in tobacco, produces the siderophore pyoverdine, which serves as a key virulence factor by facilitating bacterial growth on the host plants [58]. Taguchi and colleagues demonstrated that the mutants in pyoverdine biosynthesis enzyme–encoding genes exhibit low production of tabtoxin, extracellular polysaccharide, and quorum-sensing molecule acyl homoserine lactones (AHLs) but display accelerated swarming ability and increased biosurfactant production [58]. These studies have indicated that siderophores have a multifaceted role in the biological functions of phytopathogenic bacteria, extending beyond iron sequestration. However, in contrast, *Pseudomonas syringae* pv. phaseolicola 1448a, the causal agent of bean halo blight, produces two different types of siderophores, pyoverdine and achromobactin, yet neither of them appears to contribute to the virulence on bean plants [59]. The xanthomonads possess the *Xanthomonas* siderophore synthesis (*xss*) gene cluster, which is conserved in almost all members of the *Xanthomonas* genus and displays homology with the *pvs* gene cluster that encodes vibrioferrin in the *Vibrio* group of human pathogenic bacteria [60,61,62]. The *Xanthomonas* group of phytopathogens cause disease to approximately 400 plants, including several economically important crops, such as rice, pepper, cabbage, and tomato [63]. In the members of xanthomonads, the *xss* gene cluster encodes xanthoferrin, an α-hydroxycarboxylate-type siderophore, synthesis enzymes under low-iron conditions [60,61,64]. Xanthoferrin-mediated iron uptake promotes in planta growth of *X. oryzae* pv. oryzicola (causal agent bacterial leaf streak on rice) and *X*. *campestris* pv. campestris (causal agent black rot of crucifers) and is required for optimum virulence on their respective hosts [61,65]. Conversely, the *xss* gene cluster of *X. oryzae* pv. *oryzae*, a causal agent of bacterial blight of rice, does not express inside the plant host, and xanthoferrin-mediated iron uptake is not needed for virulence [60]. However, the *feoB* gene of *X*. *oryzae* pv. oryzae expresses inside the plant, and FeoB-mediated ferrous iron uptake critically contributes to the bacterial in planta growth and virulence on rice [60].

The above research works manifest the importance of siderophore-mediated iron uptake in the virulence of several phytopathogenic bacteria. Some phytopathogenic bacteria exhibit no impact on virulence even if the siderophore-encoding genes were knocked out (Table 1). The mutants in genes associated with siderophore biosynthesis in *Agrobacterium tumefaciens* C58, *Pseudomonas syringae* pv. tomato DC3000, and *Ralstonia solanacearum* AW1 displayed virulence proficiency on par with their wild-type strains [66,67,68]. The siderophore-mediated iron uptake is an expensive process but highly efficient in nature that secures the bacterial iron requirements by scavenging iron from iron-depleted environment. For instance, the chrysobactin produced by *Dickeya dadantii* outcompetes the plant ferritins for iron binding in *Saintpaulia* leaves [69]. The phytopathogenic bacteria have evolved with multiple iron uptake systems that confer an adaptive advantage to the pathogenic lifestyle in various host conditions. Apparently, the availability of iron inside the host and the tissue habitat of phytopathogens determine the requirement of siderophores during in planta bacterial growth and virulence. The two closely related xanthomonads, *Xanthomonas oryzae* pv. oryzae and *Xanthomonas oryzae* pv. oryzicola, share the common host rice but colonize the xylem vessel and mesophyll apoplast, respectively. The lifestyle in different habitats and the iron constituents could be attributed to the requirement of ferrous uptake through the FeoB transport system and siderophore-mediated ferric-iron uptake for the optimum virulence of *Xanthomonas oryzae* pv. oryzae and *Xanthomonas oryzae* pv. oryzicola, respectively. *Pseudomonas syringae* pv. tomato DC3000 has shown the ability to access iron from ferric citrate, but it does not contribute to pathogenicity [67].

### 2.2. Phytopathogenic Microbial Iron Storage and Virulence

As described in the introduction section, iron constitutes an essential part of several metalloproteins, including proteins containing iron–sulfur (Fe-S) clusters (Figure 1). Phytopathogenic bacteria, such as *Dickeya dadantii*, encode SufABCDSE, which is involved in the biosynthesis of Fe–S clusters under oxidative stress and contributes to virulence [73,74]. However, bacteria also widely encode specific iron storage proteins, bacterioferritin (Bfr), and bacterial ferritin (Ftn), to maintain cellular iron homeostasis. Both Bfrs and Ftns assemble from 24 identical or similar subunits of ~19 kDa into spherical structures (~120 Å diameter) of ~450 kDa with a large hollow center (~80 Å inner diameter) [75,76]. The hollow center stores around 2000 iron atoms in the form of ferric-hydroxyphosphate core [75]. Despite being similar in fold and quaternary structure, bacterial Ftns and Bfrs vary in heme content (only Bfr contains heme), composition, electrostatic properties of the pores, and binding affinity of a few crucial residues [75,76]. In *Dickeya dadantii*, *bfr* and *ftnA* encode the iron storage proteins bacterioferritin and bacterial ferritins, respectively [77]. Interestingly, the *bfr* mutant did not show a difference in growth under low iron or intracellular iron content but exhibited delayed appearance maceration symptoms on the host plant [77]. However, knocking out *ftnA* in *Dickeya dadantii* resulted in impaired growth under low iron, more sensitivity to oxidative stress, and reduced virulence on African violets [77]. Bacterioferritin-mediated iron storage in the phytopathogen *Agrobacterium tumefaciens* is required for tolerance against H_2_O_2_ exposure, maintaining intracellular iron at an optimum level, growth under low iron, and full virulence on the host plant [78]. The triple deletion of bacterioferritin-like protein encoding genes (Δ*yciE* Δ*yciF* Δ*XC_3754*) in an operon of *Xanthomonas campestris* pv. campestris 8004 resulted in lower intracellular iron content than the wild-type strain [14].

### 2.3. Transcription Regulation of Phytopathogenic Microbial Iron Homeostasis and Virulence

Iron homeostasis in numerous bacterial species is tightly regulated through the iron-responsive transcriptional regulator, ferric uptake regulator (Fur), whose function is dependent on the availability of Fe^2+^. The Fur dimer and its corepressor Fe^2+^ form the holo–Fur complex in high iron conditions, which binds to the conserved fur-box located upstream of target genes, including those encoding siderophore biosynthesis and iron uptake proteins, to actively suppress gene expression. In contrast, during low-iron conditions, Fe^2+^ dissociates from Fur, resulting in the formation of apo-Fur, which in turn dissociates from the promoter regions of target genes. This dissociation of apo-Fur enables RNA polymerase to interact with the promoter and initiate gene expression (Figure 2). The mutant strain of *Dickeya dadantii* lacking functional Fur exhibited growth deficiencies in both minimal and rich media, yet did not exhibit a discernible difference in growth under low-iron conditions [79]. Furthermore, Franza et al. demonstrated that the *fur* mutant of *Dickeya dadantii* displays the constitutive expression of high-affinity iron transport systems and reduced virulence on African violet [79]. The *fur* mutant strain of *Xanthomonas oryzae* pv. oryzae demonstrated a heightened sensitivity to the metalloantibiotic streptonigrin, implying an elevated intracellular iron concentration. Additionally, this strain exhibited suboptimal growth on nutrient-rich media, reduced catalase activity, hypersensitivity to hydrogen peroxide, and decreased virulence on rice [80]. The *fur* mutant strain of *Xanthomonas campestris* pv. campestris similarly displays an increased intracellular iron content, constitutive overproduction of siderophores, upregulated expression of iron transport genes, heightened tolerance to peroxide toxicity, and decreased virulence on the host plant [81]. In conjunction with reduced swarming motility, the *fur* mutant strain of *Pseudomonas syringae* pv. tabaci 11,528 displays constitutive production of siderophores, virulence deficiency, diminished production of tabtoxin, and reduced quantities of the quorum-sensing molecule N-acyl homoserine lactones, in addition to its already-characterized slow-growth phenotype [82]. The regulatory interdependence between iron and quorum sensing is governed in an atypical manner among the group of phytopathogenic bacteria known as xanthomonads, as elucidated in a recent review by Pandey and Chatterjee [25].

The *Xanthomonas* iron-binding regulator (XibR) binds to Fe^3+^ iron and regulates the production of siderophores, motility, and chemotaxis in *Xanthomonas campestris* pv. campestris [14]. The XibR encoding gene is conserved across xanthomonads, and homologs are also present in *Pseudoxanthomonas dokdonensis*, *Bordetella bronchiseptica*, and *Lysobacter* sp. URHA0019. Under iron-replete conditions, XibR binds to the promoter region of genes involved in siderophore synthesis, thereby repressing their expression. XibR plays a positive regulatory role in iron storage and uptake, as well as in chemotaxis and motility, while negatively regulating siderophore production. It is required for optimal virulence on the host plant [14]. The two-component system VgrS/VgrR in *Xanthomonas campestris* pv. campestris senses iron and facilitates bacterial adaptation under low-iron conditions [83]. The membrane-bound histidine kinase receptor VgrS and its cognate sensor VgrR respond to periplasmic and intracellular iron levels, respectively. Wang et al. further reported that lowering the iron level in the periplasmic regions leads to VgrS autophosphorylation. Subsequently, phosphotransfer occurs to VgrR, which directly or indirectly affects several factors involved in iron uptake, signal transduction, detoxification, cell division, and cellular metabolism [83].

### 2.4. sRNA-Mediated Regulation of Microbial Iron Homeostasis

Small RNAs (sRNAs), which are 50–400 nucleotides in length, play a crucial role as post-transcriptional regulators in prokaryotes. They are required for the fine regulation of various cellular processes, such as carbon metabolism, iron homeostasis, stress responses, motility, chemotaxis, biofilm formation, quorum sensing, and virulence. Through short base pairings, sRNAs fine-tune the stability and translation efficiency of target mRNAs. One extensively studied sRNA is RyhB in *E. coli*, which contributes to maintaining cellular iron homeostasis by regulating multiple iron utilization and transport genes [84,85]. The sRNAs regulate virulence-associated functions and stress response in phytopathogenic bacteria, such as *Pseudomonas*, *Xanthomonas*, *Agrobacterium*, and *Pectobacterium* [86,87,88,89,90]. The sRNA DsRNA-Xoo4 of *Xanthomonas* oryzae pv. oryzae [91] and sX13 of *Xanthomonas campestris* pv. vesicatoria [92,93] regulate the expression of TonB. However, further extensive investigations are required to fully understand the regulation of iron homeostasis by sRNAs in phytopathogenic microbes.

## 3. Interplay between Iron Homeostasis and Plant Immune Response

Iron is an essentially required element for several vital cellular processes of plants. Plants employ two different strategies to uptake the iron through the roots. One in nongrass plants (e.g., *Arabidopsis thaliana*), iron starvation activates a cascade of signaling reaction to release protons and phenolics in rhizosphere, which lowers the pH and solubilizes ferric iron. The ferric reductases, such as ferric reduction oxidase 2 (FRO_2_) and ferric chelate reductase (FCR), reduce ferric iron to ferrous iron, which is further transported inside the roots through a specific transporter (IRT1). Another strategy in grass plants (e.g., barley, rice), iron starvation induces the synthesis and release of the iron-chelating compounds phytosiderophores (e.g., mugineic acid) in rhizosphere. Phytosiderophore–Ferric iron complexes are transported inside the root cells by specific transporters, such as YS1 and YSL. Further, iron is distributed to the sink tissues and utilized in the formation of enzyme cofactors, etc., or stored in vacuoles or complexed with ferritins. The mechanisms and regulations of iron homeostasis in plants have been extensively reviewed in recent years [94,95,96,97,98,99]. Here, we focus the discussion on recent advances in the understanding of plant iron homeostasis in the context of microbial infections and plant host responses.

### 3.1. Bacterial Effectors Influencing Plant Iron Homeostasis

**Figure 3:**
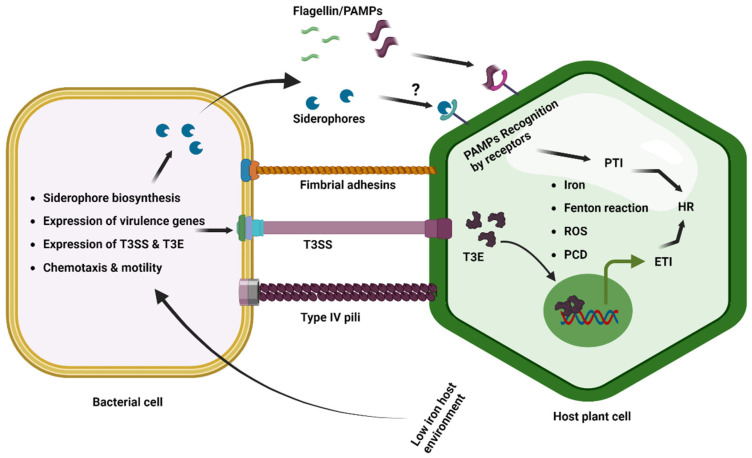
**Iron in plant–phytopathogenic bacterial interactions.** The host and phytopathogenic bacteria compete for iron resources during the in planta infection process and colonization. Plants limit the availability of iron for bacterial pathogens by transporting iron into the vacuole and sequestering it into ferritins. This results in low-iron conditions for bacteria, which trigger the induced expression of iron uptake genes and siderophore biosynthesis to obtain iron from the iron-depleted host environment. Low-iron conditions also induce bacterial motility and chemotaxis, as well as the expression of virulence genes, T3SS, and effectors. However, the response to low iron varies among bacterial pathogens. PAMP-induced PTI and effector-triggered ETI can cause the HR, which restricts bacterial growth. The siderophore pseudobactin has also been reported as a potential PAMP in *Arabidopsis*. Excess iron generates ROS via Fenton’s reaction, which triggers programmed cell death at low levels and necrosis at a threshold level.

Initially, host plants detect the microbial/pathogen-associated molecular patterns (MAMPs/PAMPs) common to many classes of microbes/pathogens to trigger PAMP-triggered immunity (PTI). Then the pathogens inject effectors in host plants to suppress the PTI that facilitates the successful disease development (Figure 3) [100]. The phytopathogenic bacterium *Pseudomonas syringae* pv. tomato DC3000 delivers an effector, AvrRps4, inside the plants and interacts with the plant iron sensor protein BRUTUS [101]. The E3 ligase BRUTUS (BTS) is an iron sensor of plants that suppresses iron deficiency response by the ubiquitin-mediated degradation of the PYE-like (PYEL) proteins IAA-LEUCINE RESISTANT3 (ILR3), bHLH105 (basic helix–loop–helix family protein), and bHLH115 through ubiquitin-mediated degradation [102,103]. The PYE-like (PYEL) proteins IAA-LEUCINE RESISTANT3 (ILR3), bHLH105, and bHLH115 are known to be induced under iron-deficient conditions and improve the iron level of plants [102,104]. The *Pseudomonas syringae* pv. tomato DC3000 effector AvrRps4 interacts with BRUTUS and induces iron accumulation in *Arabidopsis thaliana*, which potentially facilitates bacterial iron uptake and proliferation [101].

The evidence suggests that the microbial siderophores act as effector and induce plant immunity along with manipulating the host iron homeostasis [105,106,107]. The application of iron-free achromobactin and chrysobactin activates the salicylic-acid-mediated signaling pathway in *Arabidopsis thaliana* [105]. Earlier, Dellagi and colleagues demonstrated that the siderophore null mutant strain of *Dickeya dadantii* displays a compromised expression of the ferritin-encoding *AtFer1* gene in *Arabidopsis thaliana* [106]. Further, they showed that the infiltration of iron-free chrysobactin and desferrioxamine siderophores to *Arabidopsis thaliana* strongly increased AtFer1 expression. The ferritin accumulation during *Dickeya dadantii* infection appeared to be a siderophore-triggered basal defense mechanism in the host plant [106]. In another study, desferrioxamine siderophore infiltration in *Arabidopsis thaliana* resulted in the induced expression of the set of defense-related genes, accumulation of salicylic acid and jasmonic acid in leaves, and significantly higher callose deposition [107]. Several studies provided clear evidence of the modulation of host plant immune responses by siderophores. However, the potential external membrane receptors or internal receptors and the consequent cascade of reactions to induce host immune response needs more extensive investigations. 

### 3.2. Plant Immune Response Influencing Microbial Iron Homeostasis

Microbial pathogens have evolved diverse strategies to manipulate host iron homeostasis for their own iron acquisition, which in turn has driven the evolution of intricate mechanisms in plants that target microbial iron homeostasis. The expression of the sigma factor, *pvdS*, responsible for regulating the biosynthesis of pyoverdine siderophores and supporting the in planta growth of *Pseudomonas syringae* pv. tomato DC3000, was found to be suppressed as a result of an AvrRpt2-triggered ETI response [108]. Among six clusters of host-genotype-dependent DEGs identified in the AvrRpt2 of *Pseudomonas syringae* pv. tomato DC3000, the siderophore biosynthesis genes were induced within *Arabidopsis thaliana* but suppressed by the activation of both PTI and ETI [108]. The bacterial genes that are implicated in the iron acquisition pathway demonstrate the highest levels of expression in planta at 6 h postinoculation, followed by a substantial reduction in expression at 48 h postinoculation. Despite this decrease, the expression levels at 48 h postinoculation remain higher than those observed during in vitro growth conditions [109]. The findings suggest that the PTI and ETI responses in *Arabidopsis thaliana* can suppress the expression of iron starvation genes in the phytopathogenic bacterium *Pseudomonas syringae* pv. tomato DC3000. However, despite this inhibition, the bacteria retain the capacity to express these genes to a certain extent in order to acquire the necessary iron for a successful disease development.

As discussed in the previous sections, microbial pathogens and host plants undergo a complex and dynamic competition for the acquisition of iron resources. The plant host actively seeks to sequester iron, limiting its availability to the invading microbial pathogens, with the ultimate goal of curbing their growth and survival. This intricate strategy constitutes a critical component of the host response, which helps to thwart the pathogenic challenge posed by phytopathogenic microorganisms. Plants have a natural mechanism to store iron, which involves encoding ferritin proteins. Ferritin serves as an iron storage protein in plants, and its expression is regulated by various internal and external factors. Multiple studies have demonstrated the upregulation of genes encoding ferritin proteins in various plant species as a result of pathogenic attacks, including but not limited to *Arabidopsis thaliana* in response to *Dickeya dadantii* [106] and potato against *Streptomyces scabiei* infestations [110]. The plants have developed an intricate system of regulation for the expression of ferritin, a protein that stores iron, which is precisely controlled during bacterial infections to potentially impact the bacterial iron homeostasis [111]. The ethylene response factor109 (*erf109*) mutations in *Arabidopsis thaliana* caused significant changes in gene expression, with an increased expression of genes related to iron homeostasis (bHLH38, bHLH39, bHLH101, NAS4, and FER1) and a decreased expression of defense-related genes (CML37, WRKY40, ERF13, and EXO70B2), while *erf109* leaves showed elevated iron levels in both iron-sufficient and iron-deficient conditions, suggesting a potential involvement of ERF109 in regulating iron metabolism [112]. The natural resistance-associated macrophage protein (NRAMP) metal ion transporter plays a crucial role in the innate immunity of animal macrophages targeted by intracellular bacterial pathogens [113]. Segond et al. reported that AtNRAMP3, a homolog of NRAMP in *Arabidopsis*, is expressed at higher levels in leaves infected with the bacterial pathogens *Pseudomonas syringae* and *Erwinia chrysanthemi* [114]. They showed, using single and double mutants of *nramp3* and *nramp4* and ectopically expressing either of them, that AtNRAMP3 and, up to some extent, AtNRAMP4 contribute to the resistance against *Erwinia chrysanthemi* infection [114]. The susceptibility of the *nramp3nramp4* double mutant is linked to reduced levels of ROS and the iron storage protein AtFER1 ferritin, which is known to provide resistance against microbial infections in *Arabidopsis* [114].

### 3.3. Iron Availability Influencing Microbial Pathogenicity and Plant Immune Response

The indispensable role of iron in plant–phytopathogenic microbe interactions is further supported by the visible expression of virulence functions by microbes and the resulting immune responses mounted by the host, both of which are closely linked to the levels of iron available in their respective environments. For instance, iron-deficient *Arabidopsis thaliana* exhibits reduced symptom severity, bacterial fitness, and the expression of bacterial pectate lyase-encoding genes when challenged with the bacterial phytopathogen *Dickeya dadantii* [115]. The reduced symptoms and bacterial fitness observed in iron-deficient plants also extended to the necrotrophic fungus *Botrytis cinerea*. The authors demonstrated that the plant’s iron status can influence the outcome of an infection by affecting both the pathogen’s virulence and the plant’s defense response [115]. The wild-type *Arabidopsis* Col-0 plants with iron deficiency exhibit heightened resistance to infections caused by pathogens with various lifestyles, including the necrotrophic fungus *Botrytis cinerea*, the hemibiotrophic bacterium *Pseudomonas syringae* pv. tomato DC3000, and the obligate biotrophic oomycete *Hyaloperonospora arabidopsidis* [116]. The iron-deficiency-induced systemic response provides defense against pathogenic infection by the involvement of ethylene and salicylic acid signaling pathways. Disturbance in iron homeostasis within the plant, through either inducing iron starvation stress or subjecting it to other nonhomeostatic conditions, is found to have a profound effect on the plant’s immune system. In fact, such disturbances act as a trigger, priming the plant’s immune system for an enhanced defense response [116].The deposition of ferritin, which is recognized for its ability to capture iron and minimize the iron availability, in the foliage of genetically modified tobacco displayed resistance to necrotic injury that resulted from viral (tobacco necrosis virus) and fungal (*Alternaria alternata*, *Botrytis cinerea*) infections [117].

On the contrary, certain phytopathogenic bacterial species possess the capacity to detect suboptimal concentrations of iron within host plants, which elicits the activation of virulence-associated mechanisms facilitating the efficacious establishment of the infection. In response to the limited iron availability, *Xanthomonas campestris* pv. campestris displays an induced expression of multiple virulence factors, including the type 3 secretion system (T3SS) and type 3 effectors (T3E), which could be repressed by exogenous iron supplementation [118]. AN exogenous supplementation of iron at a concentration of 100 µM FeS4 was observed to exert a significant suppressive effect on the disease development ability of *Xanthomonas campestris* pv. campestris on cabbage plants, while a complete abrogation of symptom development was achieved with a relatively high iron supplementation (250 µM FeS4). Interestingly, this modulation of pathogenicity was not accompanied by any significant impact on the in planta bacterial population dynamics, indicating that iron availability may specifically regulate virulence-associated functions rather than bacterial fitness in the plant host [118]. Interestingly, the *Xanthomonas oryzae* pv. oryzicola pathovars also exhibit an induced expression of *hrp* genes under in vitro low iron, albeit to a lesser extent in comparison with *Xanthomonas campestris* pv. campestris. However, *Xanthomonas oryzae* pv. oryzae fails to demonstrate any significant induction of *hrp* gene expression under in vitro low iron [118]. The results of the study indicate that different pathogenic bacteria belonging to the same group can adapt in various ways, depending on the host plant and tissue habitat they infect, as well as the extent of iron availability in their environment. This diversity in adaptation highlights the complexity of interactions between bacterial pathogens and their plant hosts, as well as the importance of available iron in shaping these interactions. A recent study demonstrated that the application of bioengineered chitosan–iron nanocomposites can effectively mitigate the severity of *Xanthomonas oryzae* pv. oryzae caused bacterial leaf blight disease in rice, primarily attributable to their capacity to modulate the expression of host defense genes and cellular physiology, as well as their inhibitory effects on bacterial proliferation [119].

### 3.4. Ferroptotic Cell Death (FCD)

Recent studies suggest that iron, acting as a catalyst, triggers the initiation of ferroptosis, a nonapoptotic programmed cell death pathway, through the generation of reactive oxygen species (ROS), particularly lipid peroxides. This phenomenon was initially identified in animals and has also been reported in plants as a response to heat stress and pathogenic interactions [120,121,122]. During an incompatible interaction between rice plants and the avirulent strain *Magnaporthe oryzae* INA 168, a notable elevation in intracellular ferric iron and ROS accumulation is observed. This surge in ferric iron and ROS levels subsequently triggers FCD, leading to the inhibition of pathogen growth. Conversely, in a compatible interaction with the virulent strain *Magnaporthe oryzae* PO6-6, there is no significant increase in iron accumulation within the rice plants, thereby facilitating the efficient growth and proliferation of the pathogen [121]. Remarkably, rice ferric iron storage protein ferritin 2 (OsFER2) plays a crucial role in iron–ROS–mediated FCD as a defense response against avirulent strain *Magnaporthe oryzae* INA 168 infection [123]. Furthermore, a recent study involving iTRAQ-based quantitative proteomics analysis and the use of iron chelators has indicated the occurrence of ferroptosis-like cell death in *Nicotiana benthamiana* plants infected with the highly virulent tobacco mosaic virus mutant 24A+UPD [124].

## 4. Future Perspectives

Under both biotic and abiotic stress, the health of plants can be further compromised due to the production of ROS, triggered by excess intracellular iron via Fenton’s reaction and consequent oxidative burst. The dynamic evolutionary interplay between microbes and plants underscores the central role of iron in the struggle for survival and highlights the importance of understanding the molecular mechanisms underlying host–pathogen interactions. However, research on the role of iron in plant–phytopathogenic microbe interactions lags far behind that of animal host–pathogenic microbe associations. Recent studies have highlighted the potential of iron as a therapeutic tool to improve plant health under pathogenic attack. For example, the application of iron-containing bioengineered nanoparticles has been shown to alleviate bacterial leaf blight disease in rice, while direct administration of iron can completely shut down the expression of virulence genes in *Xanthomonas campestris* pv. campestris and suppress symptom appearance on cabbage, demonstrating the therapeutic potential of iron [118,119]. Alternatively, the occurrence of low iron levels and the subsequent iron deficiency response actually stimulates the host’s defense against pathogen attacks. Recent studies have provided a clearer understanding of induced systemic resistance (ISR), a phenomenon whereby microbes present in the rhizosphere trigger a response to iron deficiency within plant roots. This response plays a vital role in enhancing plant defense mechanisms, thereby bolstering their ability to combat various pathogenic infections [125,126]. The rhizobacterium *Pseudomonas simiae* WCS417, which colonizes the roots of plants, exerts a stimulating effect on plant growth and confers enhanced resistance against a range of diseases. In the model plant *Arabidopsis thaliana*, WCS417 elicits a root response similar to that observed during iron (Fe) starvation, thereby activating specific genes involved in the response to iron deficiency, namely, MYB72 and IRT1. Even under normal iron conditions, WCS417 transiently induces an iron deficiency response in the roots, resulting in an increase in both the total iron content and the fresh weight of the shoots. The induction of the iron deficiency response in the roots by WCS417 is governed by a signaling mechanism from the shoots to the roots, which operates independently of the iron status of the leaves and the phloem-specific iron transporter -*opt3* gene [126]. These studies indicate the potential for further research in the field of biofertilizers, with a focus on considering the iron content in the rhizosphere. Such research could lead to advancements in horticultural practices and disease management strategies, offering potential improvements in crop yield and health. The regulation of iron homeostasis in host organisms and phytopathogenic microbes must be carefully leveraged to achieve optimal outcomes in therapeutic applications. The interaction between microbial virulence and host defense responses, particularly in the context of iron homeostasis regulation, demonstrates significant diversity and objectively varies in different cases. Hence, the research should consider that fine-tuning the manipulation of iron homeostasis for therapeutic purposes is also crucial while taking into account the multiple roles of iron during host–microbe interactions. In addition to the availability of in planta free iron for microbial pathogens, iron plays a dual role by generating ROS as a host defense response and low-iron-induced signaling for ISR to boost immunity against pathogenic infections. The exotic disease Huanglongbing (HLB) of citrus, caused by the bacterium *Candidatus* Liberibacter asiaticus, has caused severe damage to the Florida citrus industry [127,128]. Recent studies have demonstrated that *Candidatus* Liberibacter asiaticus lacks any virulence factors that can directly cause HLB symptoms; rather, the disease results from phloem cell death and necrosis due to excessive ROS generation resulting from an overreaction of the plant’s immune response to bacterial infection [129,130]. The potential involvement of iron in the generation of ROS in this pathosystem highlights the need for further investigation to determine the extent of iron’s role. Such research may enable the development of remedial interventions that manipulate host iron levels for potential therapeutic benefit. To date, investigations into the contribution of iron to virulence have focused primarily on a limited subset of microbial phytopathogens, including *Dickeya dadantii*, *Erwinia* spp., *Pseudomonas* spp., and *Xanthomonas* spp. However, additional investigations are required not only to acquire a thorough comprehension of the molecular mechanisms underlying iron homeostasis and its role in virulence in these organisms but also in relatively less explored pathosystems, such as *Candidatus* Liberibacter asiaticus, *Phytoplasma*, *Pectobacterium*, among others.

## 5. Conclusions

Iron is a crucial component of plant nutrition, but its excess can generate ROS and induce stress. Plant–microbe interactions are shaped by their strategies to access and regulate iron resources. Various studies have demonstrated the importance of iron in regulating the virulence of phytopathogenic microbes and the defense responses of host plants. Both pathogens and plants evolved mechanisms to manipulate each other’s iron homeostasis to gain advantages in the virulence war. Recent research has shown that manipulating iron levels can be a therapeutic approach for disease management. To develop effective disease management strategies and engineer disease-resistant plants, it is necessary to gain a detailed understanding of the involvement of iron in plant–microbe interactions. Biotechnological approaches for manipulating iron levels in plants may prove useful in safeguarding them against abiotic and biotic stresses. These approaches have significant potential to contribute to sustainable agricultural practices, which are increasingly important given the changing climate and growing global population.

## Figures and Tables

**Table 1:** Iron-chelating compounds produced by different phytopathogenic bacteria, along with their respective roles in promoting virulence.

S. No.	Phytopathogenic Bacteria	Iron Chelator	Role in Virulence	Reference
01.	*Dickeya dadantii* (syn. *Erwinia chrysanthemi*)	Chrysobactin, achromobactin	Required for optimum virulence	[51,52]
02.	*Dickeya dadantii*	IbpS	Required for optimum virulence	[53]
03.	*Erwinia amylovora*	Desferrioxamine (DFO)	Required for optimum virulence	[54]
04.	*Erwinia carotovora* subsp. carotovora	Aerobactin, Chrysobactin	Not required for virulence	[56,57,70]
05.	*Pseudomonas syringae* pv. tabaci 6605	Pyoverdine	Required for optimum virulence	[58]
06.	*Pseudomonas syringae* pv. phaseolicola 1448a	Pyoverdine, achromobactin	Not required for virulence	[59]
07.	*Pseudomonas syringae* pv. syringae B301D	Pyoverdine	Not required for virulence	[71]
08.	*Pseudomonas syringae* pv. tomato DC3000	Pyoverdine, yersiniabactin	Not required for virulence	[67]
09.	*Xanthomonas campestris* pv. campestris 8004	Xanthoferrin	Required for optimum virulence	[61]
10.	*Xanthomonas oryzae* pv. oryzae BXO1	Xanthoferrin	Not required for virulence	[60,64]
11.	*Xanthomonas oryzae* pv. oryzicola BXOR1	Xanthoferrin	Required for optimum virulence	[64,65]
12.	*Xanthomonas campestris* pv. campestris 8004	Cyclic β-(1,2)-glucans	Required for optimum virulence	[33]
13.	*Agrobacterium tumefaciens* C58	Unknown iron chelator	Not required for virulence	[66]
14.	*Agrobacterium* tumefaciens strain B6	Agrobactin	Not required for virulence	[72]
15.	Ralstonia solanacearum AW1	Staphyloferrin B	Not required for virulence	[68]

## Data Availability

Data is contained within the article.
